# Exosomal circ_0088300 Derived From Cancer-Associated Fibroblasts Acts as a miR-1305 Sponge and Promotes Gastric Carcinoma Cell Tumorigenesis

**DOI:** 10.3389/fcell.2021.676319

**Published:** 2021-05-26

**Authors:** Hao Shi, Shan Huang, Mingde Qin, Xiaofeng Xue, Xingpo Guo, Linhua Jiang, Han Hong, Jian Fang, Ling Gao

**Affiliations:** ^1^Department of General Surgery, The First Affiliated Hospital of Soochow University, Suzhou, China; ^2^Department of Pathology, The First Affiliated Hospital of Soochow University, Suzhou, China; ^3^The Stem Cell and Biomedical Material Key Laboratory of Jiangsu Province (The State Key Laboratory Incubation Base), Soochow University, Suzhou, China; ^4^Department of Hepato-Pancreato-Biliary Surgery, Suzhou Municipal Hospital, The Affiliated Suzhou Hospital of Nanjing Medical University, Suzhou, China; ^5^Department of General Surgery, Zhangjiagang Hospital Affiliated to Soochow University, Zhangjiagang, China

**Keywords:** exosomes, circularRNA_0088300, cancer-associated fibroblasts, microRNA-1305, gastric carcinoma, tumorigenesis

## Abstract

Cancer-associated fibroblast (CAF)-derived exosomes play a major role in gastric carcinoma (GC) tumorigenesis. However, the mechanism behind the activity of circular RNAs in CAF-derived exosomes in GC remains unclear. In the present study, we identified differentially expressed circ_0088300 in GC tissues and plasma exosomes. We found that CAFs delivered functional circ_0088300 to GC tumor cells via exosomes and promoted the proliferation, migration and invasion abilities of GC cells. Furthermore, we demonstrated that circ_0088300 packaging into exosomes was driven by KHDRBS3. In addition, we verified that circ_0088300 served as a sponge that directly targeted miR-1305 and promoted GC cell proliferation, migration and invasion. Finally, the JAK/STAT signaling pathway was found to be involved in the circ_0088300/miR-1305 axis, which accelerates GC tumorigenesis. In conclusion, our results indicated a previously unknown regulatory pathway in which exosomal circ_0088300 derived from CAFs acts as a sponge of miR-1305 and promotes GC cell proliferation, migration and invasion; these data identify a potential biomarker and novel therapeutic target for GC in the future.

## Introduction

Gastric carcinoma (GC) is one of the most aggressive and common tumors worldwide, particularly in East Asian countries (de Martel et al., 2013). Although progress has been made in the early diagnosis and comprehensive therapy of GC, the survival rate of GC patients has been worryingly high in recent years (Mihmanli et al., 2016). Indeed, the 5-year overall survival rate is 20–25% in patients who suffer from postoperative recurrence and inefficient chemotherapy (Razzak, 2013). Thus, it is urgent to explore the deep biological mechanism of GC to enable new diagnoses and therapies for GC patients.

Cancer-associated fibroblasts (CAFs), the main components of the tumor stroma, play a crucial role in the tumor microenvironment (Zhao et al., 2013). Recent studies have illustrated that the tumor microenvironment not only provides physical support for cancer cells but also manages cell-to-cell interactions, including the interaction between cancer cells and CAFs (Bu et al., 2019; Jang and Beningo, 2019). A previous study demonstrated that cancer cells induce an anti-Warburg effect in adjacent CAFs to promote cancer progression (Pavlides et al., 2009). Recently, another research group showed that energy-rich metabolites derived from CAFs support the mitochondrial respiration and anabolic metabolism of cancer cells (Shan et al., 2017). Moreover, cancer cells also induce the switching of various types of cells, including epithelial, endothelial, and smooth muscle cells, into CAFs (Xing et al., 2010). Furthermore, it has also been shown that exosomes derived from CAFs are able to increase tumor cell chemoresistance in many cancer types (Au Yeung et al., 2016; Ren et al., 2018).

Exosomes are extracellular vesicles with diameters of 30–100 nm, and they are derived from almost all cell types (Thery et al., 2002; Zhang Y. et al., 2019). Exosomes have been explored as a potential biomarker in many diseases due to their transport of proteins, mRNAs, miRNAs and circRNAs into target cells, which plays an important role in communication between the cells (Barile and Vassalli, 2017; Mashouri et al., 2019). Circular RNAs (circRNAs) are a type of widespread endogenous non-coding RNA that can regulate gene expression and the cell cycle in mammals (Hansen et al., 2013; Memczak et al., 2013). Previous studies have shown that the presence of circRNAs in exosomes may be directed by their association with miRNAs in the cells deriving the exosomes, resulting in the transfer of their biological activity to recipient cells (Li et al., 2015; Fanale et al., 2018). In addition, it has also been demonstrated that circNRIP1 acts as a tumor promotor in GC and that exosomal circNRIP1 promotes tumor metastasis *in vivo* (Zhang X. et al., 2019). Furthermore, CAF-derived exosomes increase chemoresistance-inducing factors in recipient epithelial cells and promote proliferation and drug resistance in pancreatic cancer cells (Richards et al., 2017). However, the mechanism by which CAF-derived exosomes regulate GC tumorigenesis is not fully understood, and whether circRNAs in CAF-derived exosomes play an important role in GC still needs to be explored.

In the present study, we aimed to identify circRNAs that may be involved in the pathology of GC using GEO microarray datasets. In addition, we identified a novel circular RNA, circ_0088300, that was derived from CAFs, and we examined the detailed mechanism of circ_0088300 activity in GC progression. Furthermore, we also explored the molecular mechanisms by which exosomal circ_0088300 promotes tumorigenesis in GC cells.

## Materials and Methods

### Patient Samples

GC tissues and corresponding normal stomach mucosa tissues were obtained from 60 patients who were diagnosed with GC at The First Affiliated Hospital of Soochow University from July 2014 to September 2017 (detailed patient information is shown in Supplementary Excel 1). Approval for this study was provided by the Ethics Committee of The First Affiliated Hospital of Soochow University. Informed consent was obtained from each GC patient. Tissues were immersed in liquid nitrogen immediately after removal from patients and then were stored at –80°C until use. Moreover, 60 plasma samples from volunteers who underwent physical examination at the First Affiliated Hospital of Soochow University were used as healthy controls.

### Cell Culture

GES1 stomach mucosa epithelium cells and human GC cell lines of SGC7901, BGC-823, AGS, and MGC-803 cells were provided by the Cell Center of Shanghai Institutes for Biological Sciences. CAFs were isolated from GC tissues for primary culture, while normal fibroblasts (NFs) were isolated from the corresponding adjacent normal tissues. All the cells were cultured in RPMI 1640 medium except the AGS cells, which were cultured in F12K medium. Both media were supplemented with 10% fetal bovine serum (Gibco, United States), 100 U/mL streptomycin and 100 U/mL penicillin (Gibco, United States). Cells were incubated at 37°C and 5% CO_2_.

### Quantitative Real-Time PCR (qRT-PCR), Primers, siRNAs, and Probes

Total RNA was extracted from GC tissues, normal tissues, NFs, CAFs, SGC7901 cells, BGC-823 cells, AGS cells, and MGC-803 cells using TRIzol reagent (TransGen Biotech, China) according to the manufacturer’s protocol. A cDNA Reverse Transcription kit (TransGen Biotech, China) was used to generate cDNA from RNA. qRT-PCR was performed using a SYBR Green kit (Qiagen, United States) in an ABI 7900 PCR Thermal Cycler according to the manufacturer’s protocol. GAPDH was used as the endogenous control to standardize circRNA expression. The sequences of the primers, siRNAs and probes are listed in Supplementary Tables 1–3.

### Fluorescence *in situ* Hybridization (FISH)

Circ_0088300 probes were provided by Songan Biotech (Shanghai, China). The probe signals were measured by a fluorescent *in situ* hybridization kit (RiboBio, Guangzhou, China) according to the manufacturer’s protocols. The cells were treated with 4’-6-diamidino-2-phenylindole (DAPI; Sigma, United States) as a control.

### 5-Ethynyl-2’-deoxyuridine (EdU) Assay

An EdU assay kit (Abcam, United States) was used to detect DNA synthesis and cell proliferation. We seeded 1 × 10^4^ SGC-7901 or BGC-823 cells in a 96-well plate overnight and then added Edu solution (25 μM) into the wells and incubated them for 24 h. Then, 4% paraformaldehyde was applied at RT for 30 min to fix the cells. Triton X-100 (0.5%) was added for 10 min to permeabilize the cells, and then Apollo reaction solution (200 μL) was added to stain the EdU for 30 min and Hoechst 33342 (200 μL) was added to stain the nuclei. Finally, we visualized the cells under a fluorescence microscope (IX81, Olympus, Japan) to observe DNA synthesis and cell proliferation.

### Animal Studies

Four-week-old male nude mice were purchased from the National Laboratory Animal Center (Shanghai, China). The animal studies were approved by the First Affiliated Hospital of Soochow University. In total, 20 mice (*n* = 5) were injected with stable si-NC SGC-7901 cells, si-circ_0088300 SGC-7901 cells, or SGC-7901 cells that were treated with si-NC-CAF-Exo, SGC-7901 cells that were treated with si-circ_0088300-CAF-Exo and were resuspended in growth medium (150 μL) and Matrigel substrate (150 μL). The mice were injected with 4.0 mg of luciferin (Gold Biotech) in 50 μL of saline. After 1 h, tumors were detected using an IVIS@ Lumina II system (Caliper Life Sciences, Hopkinton, MA). The animals were sacrificed 35 days after injection, and the tumors were collected to measure the tumor volume every 7 days. The tumor volume was calculated using the following formula: volume (mm^3^) = length × width^2^/2.

### Statistical Analysis

The data are presented as the mean ± standard deviation (SD) and were analyzed using one-way analysis of variance and Tukey’s *post-hoc* multiple comparison tests. All experimental data were analyzed using SPSS 25.0. Values were considered statistically significant when *P*-values were 0.05.

## Results

### Identification of circ_0088300 as a GC Diagnosis and Prognosis Biomarker

**FIGURE 1:**
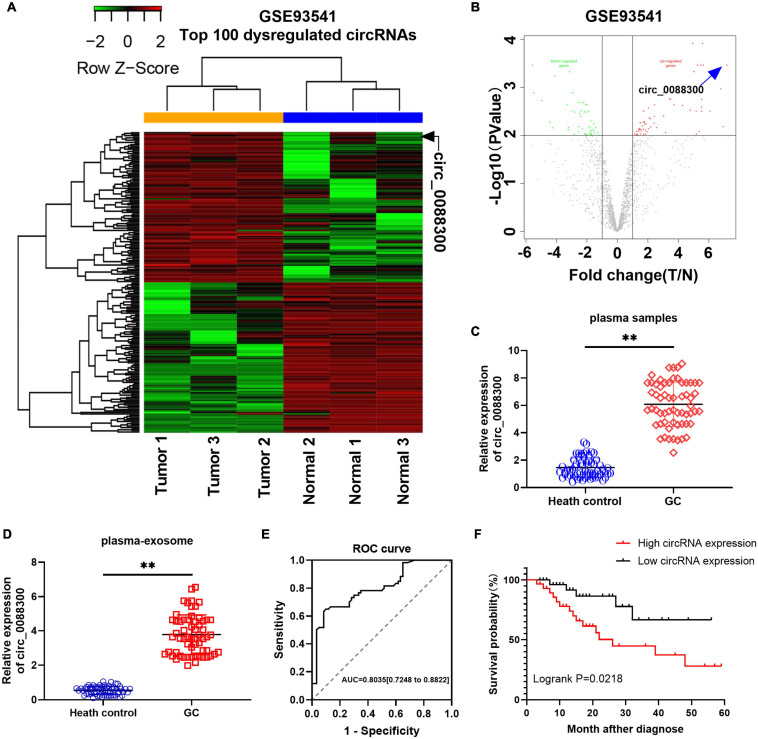
Identificationof circ_0088300 as a GC diagnosis and prognosis biomarker. **(A)** Heat map showing the top 100 dysregulated circRNAs in the GSE93541 dataset, which are displayed on a scale from green (low) to red (high); data are based on three paired human GC plasma samples and normal controls. The arrow represents the circRNA (hsa_circ_0088300) with the greatest fold changes. **(B)** Volcano plot map of the dysregulated circRNAs in the GSE93541 dataset, and the arrow represents circ_0088300 with the greatest upregulation in GC cells. **(C)** The relative expression of circ_0088300 in plasma of 60 GC patients and 60 volunteers. **(D)** The relative expression of circ_0088300 in exosomes derived from the plasma of 60 GC patients and 60 volunteers. **(E)** ROC curve of circ_0088300 in GC. **(F)** Survival curve of GC patients with highexpression (*n* = 30) of circ_0088300 or low expression (*n* = 30) of circ_0088300 (^∗∗^*P* < 0.01).

To identify the Exo-circRNAs involved in GC tumorigenesis, we first searched the GEO database with “circRNA” and “gastric cancer plasma,” and the results showed that there was only one GSE dataset (GSE93541). A clustered heat map (Figure 1A) shows the top 100 dysregulated circRNAs, and a volcano plot shows that hsa_circ_0088300 was the most upregulated (filtered by -logFC value) circRNA (Figure 1B). Then, we investigated the cir_0088300 information via a bioinformatics method (UCSC Date) and observed that circ_0088300 was produced by the PSMD5 gene, which is located at chromosome 9 (q33, 2) and consists of head-to-tail splicing from exon 2 to exon 4; the spliced length was 388 bp (Supplementary Figure 1A). PCR analysis indicated that divergent primers could produce the circular isoform of circ_0088300 with cDNA but not with genomic DNA (gDNA), while convergent primers could amplify the linear isoform of circ_0088300 from both cDNA and gDNA in GC tissues (Supplementary Figures 1B,C). Sanger sequencing results also confirmed the identity of the circular form of circ_0088300 (Supplementary Figure 1A). Moreover, qRT-PCR showed that circ_0088300 can resist RNase R, while liner mRNA can be degraded by RNase R; further, circ_0088300 was detected to be enriched in the cytoplasm instead of in the nucleus (Supplementary Figure 1D). The relative expression of circ_0088300 in the plasma samples of 60 GC patients was significantly higher than it was in the plasma samples of the 60 control patients (*P* < 0.01; Figure 1C). Then, we isolated exosomes from plasma samples, and qRT-PCR of plasma exosomes showed that circ_0088300 in GC plasmaexosomes was upregulated compared with that of normal plasmaexosomes (*P* < 0.01; Figure 1D). The ROC curve shows that the AUC = 0.7961, which means that circ_0088300 has high accuracy in the diagnosis of GC patients (Figure 1E). The survival curve also showed that GC patients who had high circ_0088300 expression had a lower survival probability after diagnosis compared with patients who had low circ_0088300 expression (Logrank *P* < 0.01; Figure 1F). Compared with the GC clinicopathologic features, the circ_0088300 levels in GC tissues corresponded to the levels in plasma exosomes (Supplementary Tables 4, 5). These results indicate that circ_0088300 can act as a GC diagnosis and prognosis biomarker.

### Exosomal Transfer of circ_0088300 From CAFs to GC Cells

**FIGURE 2:**
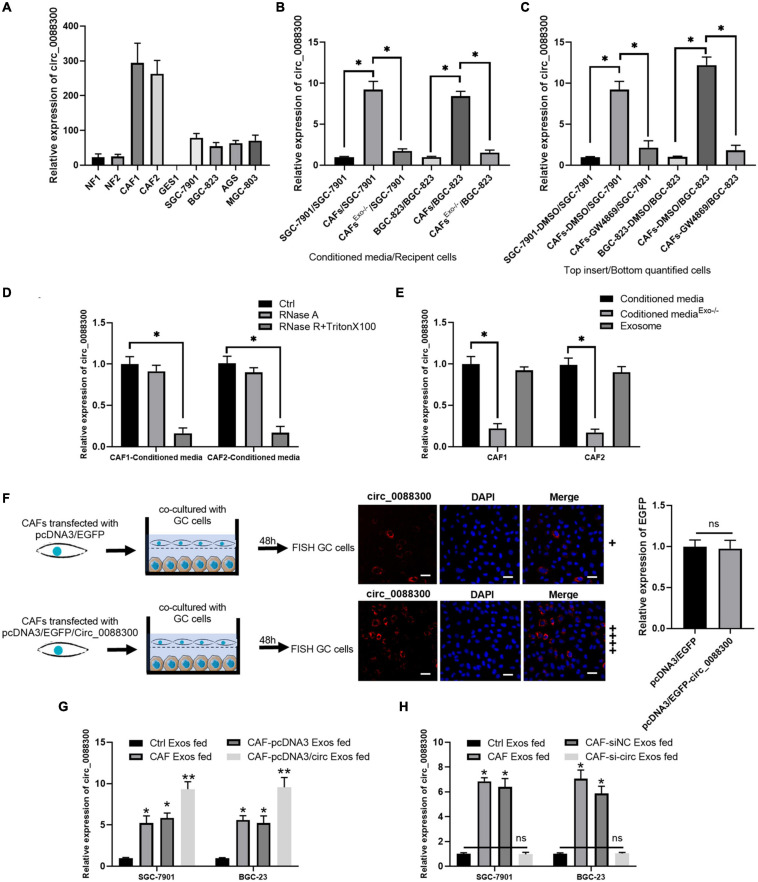
CAFs in the tumor microenvironment might increase circ_0088300 expression levels in GC cells through direct circ_0088300 transfer. **(A)** circ_0088300 expression in NFs, CAFs, normal stomach epithelial cells (GES1), and GC cell lines (SGC-7901, BGC-823, AGS, and MGC-803) was analyzed via qRT- PCR. **(B)** SGC-7901 and BGC-823 cells were incubated with control conditioned media (CM), CAF-CM, or exosome-depleted CAF-CM for 24 h, and the circ_0088300 expression level in these cells was detected by qRT-PCR. **(C)** SGC-7901 and BGC-823 cells were co-cultured with DMSO-treated SGC-7901 and BGC-823 cells, DMSO-treated CAFs, or GW4869-treated CAFs for 24 h. The circ_0088300 expression level was detected in GC cells by qRT-PCR. **(D)** qRT-PCR of circ_0088300 expression in CAF-CM treated with RNase A (2 mg/mL) independently or cotreated with Triton X-100 (0.1%) for 20 min. **(E)** qRT-PCR of circ_0088300 expression in exosomes, exosome-depleted CM, and whole CM derived from CAFs. **(F)** CAFs transfected with pcDNA3/EGFP/circ_0088300 were cocultured with GC cells for 48 h. Fluorescence microscopy was used to detect red fluorescent signals in GC cells (scale bar = 50 μm), and the expression of EGFP between two groups was not statistically different. **(G)** circ_0088300 expression in SGC-7901 and BGC-823 cells was detected by qRT-PCR at 24 h after incubation with exosomes (25 μg/mL) derived from GC cells (Ctrl Exos), CAFs, or CAFs transfected with or without circ_0088300 mimics. **(H)** circ_0088300 expression in SGC-7901 and BGC-823 cells was detected by qRT-PCR at 24 h after incubation with exosomes (25 μg/mL) derived from GC cells (Ctrl Exos), CAFs, or CAFs transfected with or without circ_0088300 siRNA (ns, no significant difference; *P < 0.05; **P < 0.01).

To identify the source of circ_0088300, we evaluated its expression level in various cell lines, SGC-7901, BGC-823, AGS, and MGC-803, as well as in primary NFs and CAFs, and the normal stomach mucosa epithelium cell line GES1. Two pairs of NFs and CAFs were isolated from two GC tissues and adjacent normal tissues, and the morphology and Western blot results revealed that they exhibited the typical characteristics of NFs and CAFs (Supplementary Figures 2A,B). The results further showed that the relative expression of circ_0088300 in CAFs was significantly higher than it was in other cells (*P* < 0.01; Figure 2A). These results suggest that CAFs in the tumor microenvironment might increase circ_0088300 expression levels in GC cells through direct circ_0088300 transfer.

Exosomes have been indicated as transporters for cell communication, so we isolated exosomes derived from CAFs and identified them via TEM and Western blot (Supplementary Figures 2C,D). To investigate whether circ_0088300 is transferred from CAFs to tumor cells via exosomes, GC cells (SGC-7901 and BGC-823) were incubated with CAF-conditioned media (CM) and exosome-depleted CAF-CM. We observed that GC cells cultured in CAF-CM expressed a higher level of circ_0088300, while circ_0088300 expression in GC cells was significantly downregulated when exosomes derived from CAFs were depleted physically or pharmacologically (*P* < 0.01; Figures 2B,C). Moreover, the levels of circ_0088300 in CAF-CM were unvaried upon RNase A treatment, but they were substantially decreased when exposed to RNase A + Triton X-100 (Figure 2D), indicating that extracellular circ_0088300 was mostly contained within the membrane and not directly released. The levels of circ_0088300 were almost indistinguishable in exosomes and normal CAF-CM (Figure 2E). CAFs transfected with pcDNA3/EGFP/Circ_0088300 were cocultured with GC cells for 48 h, and the qRT-PCR results showed that the transfection efficiency was adequate (Supplementary Figures 3A,B). The FISH GC cell results, which were observed via fluorescence microscopy, showed that circ_0088300 was transferred from CAFs to GC cells (Figure 2F). Additionally, intracellular circ_0088300 levels were significantly upregulated through incubation with exosomes from CAFs in which circ_0088300 was overexpressed as opposed to CAFs in which circ_0088300 was inhibited (Figures 2G,H). These results indicated that circ_0088300 transferred from CAFs to GC cells via exosomes.

### Circ_0088300 Loading Into Exosomes Is Mediated by KHDRBS3

**FIGURE 3:**
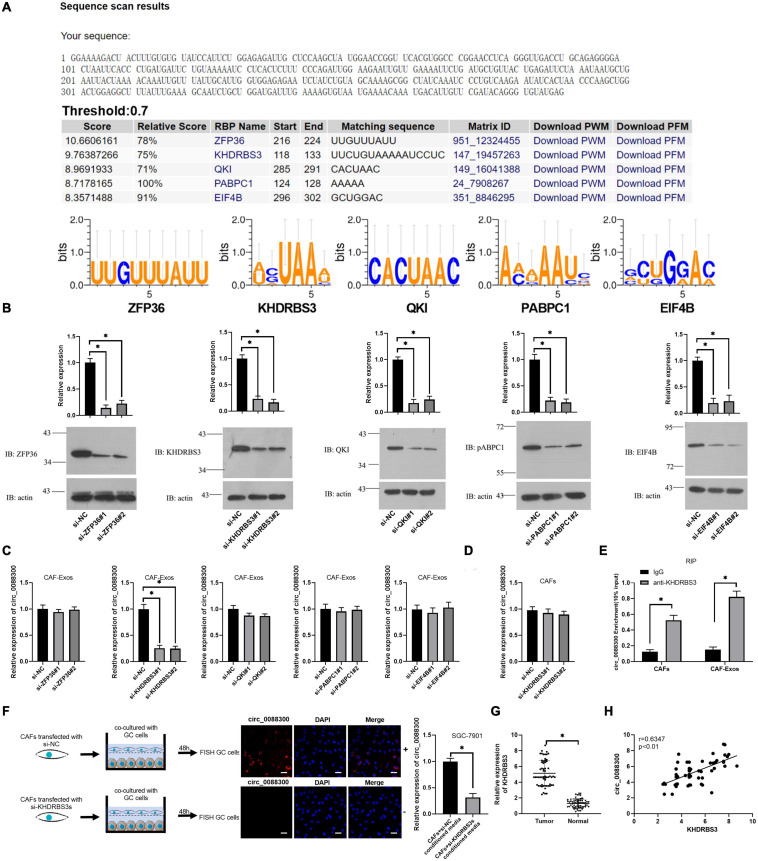
KHDRBS3 mediates circ_0088300 packaging into CAF-derived exosomes. **(A)** A specific interaction between RBP motifs and circ_0088300 was predicted via RBPDB analysis (threshold = 0.7). **(B)** Western blot results showing ZFP36, KHDRBS3, PABPC1, QKI, and EIF4B expression levels in CAFs at 48 h after transfection with specific siRNAs. **(C)** qRT-PCR analysis of the expression of circ_0088300 in CAF-derived exosomes in different cell groups transfected with specific siRNAs targeting ZFP36, KHDRBS3, PABPC1, QKI, and EIF4B. **(D)** the expression of circ_0088300 in CAFs transfected with specific siRNAs targeting KHDRBS3 was measured by qRT-PCR. **(E)** RIP assays with an anti-KHDRBS3 antibody (or IgG as a control) were performed on the cell or exosomal lysates from CAFs. Circ_0088300 levels in immunoprecipitated samples were determined by qRT-PCR and are reported as percentages with respect to the input sample (% input). **(F)** GC cells were cocultured with CAFs transfected with pcDNA3/EGFP/circ_0088300 and specific siRNAs targeting KHDRBS3 for 48 h. Fluorescence microscopy was used to detect red fluorescent signals in GC cells (scale bar = 20 μm). **(G)** The expression of KHDRBS3 in GC tissues and adjacent normal tissues was analyzed by qRT-PCR. **(H)** Correlation analysis was performed between circ_0088300 expression and KHDRBS3 expression in GC tissues (*n* = 30; ^∗^*P* < 0.05).

The database of RBP specificities (RBPDB)^1^ was used to analyze the RNA binding protein motifs of circ_0088300, and the results showed that zinc finger protein 36 (ZFP36), KH RNA binding domain containing signal transduction associated 3 (KHDRBS3), quaking homolog (QKI), poly(A) binding protein cytoplasmic 1 (PABPC1), and eukaryotic translation initiation factor 4B (EIF4B) were the top five proteins predicted to bind with circ_0088300 (Figure 3A and Supplementary Excel 2). Then, we knocked down the expression of these proteins in CAFs (Figure 3B). The qRT-PCR results showed that the expression of circ_0088300 in the CAF-exosomes was downregulated when KHDRBS3 was knocked down via siRNA treatment; however, the expression of circ_0088300 in the untreated CAFs was unchanged (Figures 3C,D). These results indicated that KHDRBS3 could regulate the circ_0088300 level in exosomes. Furthermore, RNA pull-down assays showed that the 100–200 bp sequence of circ_0088300 was important for the interaction between circ_0088300 and KHDRBS3 (Supplementary Figures 4A,B). Moreover, the results of the RNA immunoprecipitation (RIP) assay indicated that the expression of circ_0088300 was increased in the KHDRBS3 antibody group compared with the IgG group in both CAFs and CAF-exosomes (Figure 3E). FISH after coculture of CAFs and GC cells revealed that circ_0088300 transfer from CAFs to GC cells by exosomes was decreased when CAFs were transfected with a KHDRBS3-siRNA (Figure 3F). In addition, we also found that the expression of KHDRBS3 was increased in GC tissues compared with adjacent normal tissues, and the expression of KHDRBS3 in GC was positively correlated with circ_0088300 levels (Figures 3G,H). In addition, a survival curve showed that GC patients who had high KHDRBS3 expression had a lower survival probability after diagnosis compared with the patients who had low expression of KHDRBS3, as determined by analysis with a KM Plot online tool (Supplementary Figure 5). These results indicated that KHDRBS3 might play a remarkable role in packaging circ_0088300 into exosomes and promote GC tumorigenesis.

### Circ_0088300 Enhances GC Cell Migration, Invasion, and Proliferation

**FIGURE 4:**
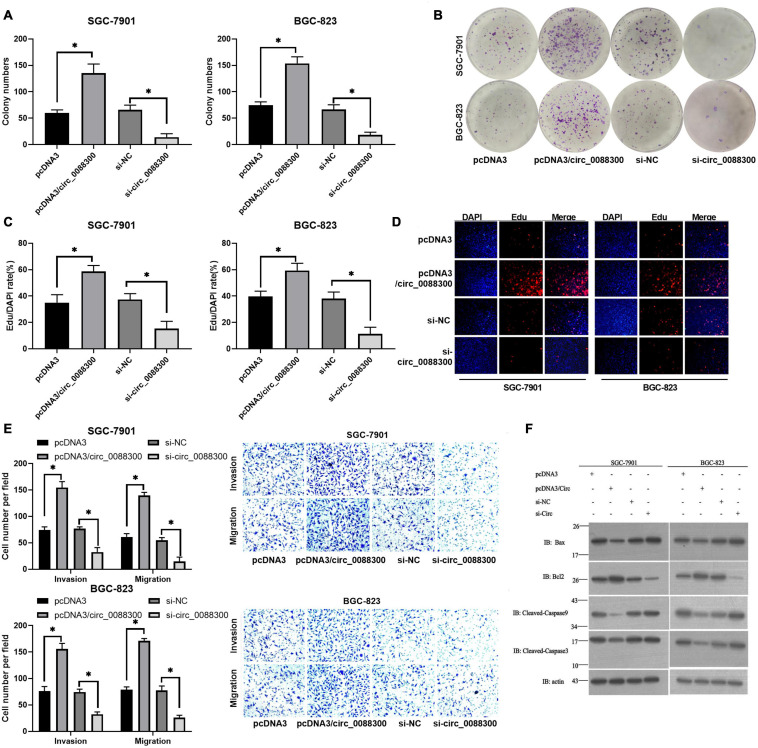
Upregulated circ_0088300 levels enhance GC cell migration, invasion and proliferation while inhibiting their apoptosis. **(A,B)** Plate colony formation assays of SGC-7901 and BGC-823 cells transfected with pcDNA3/circ_0088300 or si-circ_0088300. **(C,D)** EdU assays of SGC-7901 and BGC-823 cells transfected with pcDNA3/circ_0088300 or si-circ_0088300. **(E)** Transwell assays of SGC-7901 and BGC-823 cells transfected with pcDNA3/circ_0088300 or si-circ_0088300 were performed to evaluate cell migration and invasion abilities. **(F)** Western blot assays were used to analyze the protein expression levels of apoptosis and anti-apoptosis proteins in GC cells (^∗^*P* < 0.05).

Having proven that GC cells could take up CAF-derived exosomal circ_0088300, we next investigated whether circ_0088300 could contribute to GC cell migration, invasion and proliferation. Colony formation assays showed that circ_0088300 overexpression significantly enhanced proliferation of SGC-7901 and BGC-823 cells, while circ_0088300 knockdown decreased cell proliferation (Figures 4A,B). Simultaneously, the results showed that the percentage of EdU-positive SGC-7901 and BGC-823 cells was higher in the circ_0088300 overexpression group than in the control group, and the percentage in the circ_0088300 knockdown group was lower than that in the control group (Figures 4C,D). Moreover, the results of the Transwell assay revealed that circ_0088300 overexpression markedly enhanced migration and invasion in SGC-7901 and BGC-823 cells, while circ_0088300 knockdown significantly weakenedmigration and invasion (Figure 4E). In addition, Western blot assays showed that the relative expression of the anti-apoptosis protein Bcl-2 was upregulated and that the relative expression of the apoptosis proteins Bax, caspase 3, and caspase 9 was downregulated when circ_0088300 was overexpressed (Figure 4F). *In vivo* experiments showed that circ_0088300 knockdown in SGC-7901 cells and exosomes could reduce the tumor volume in nude rats (Supplementary Figures 6A–D). These *in vitro* and *in vivo* results indicated that circ_0088300 overexpression could enhance GC cell migration, invasion and proliferation.

### Transfer of circ_0088300 by Exosome Enhances Malignant Transformation *in vitro*

**FIGURE 5:**
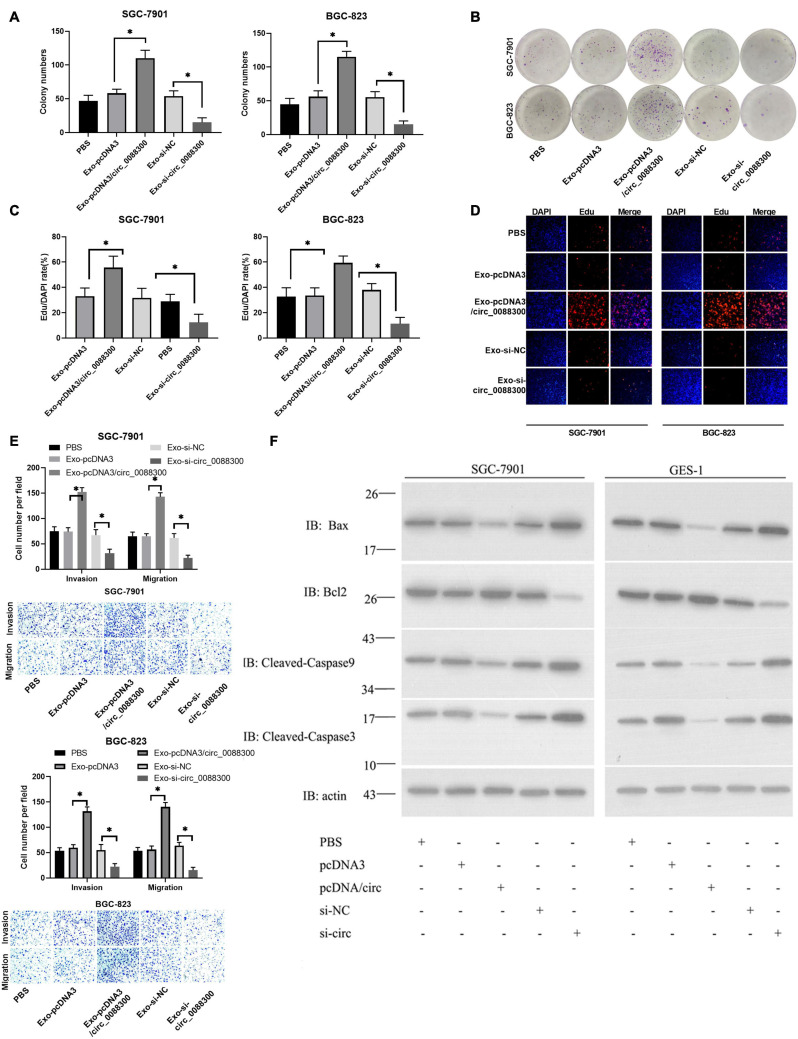
Exosomal transfer of circ_0088300 enhances malignant transformation *in vitro*. **(A,B)** Plate colony formation assay of SGC-7901 and BGC-823 cells treated with exosomes derived from CAFs transfected with pcDNA3/circ_0088300 or si-circ_0088300. **(C,D)** EdU assay of SGC-7901 and BGC-823 cells treated with exosomes derived from CAFs transfected with pcDNA3/circ_0088300 or si-circ_0088300 (scale bar = 50 μm). **(E)** Transwell assays of SGC-7901 and BGC-823 cells transfected with pcDNA3/circ_0088300 or si-circ_0088300 were performed to evaluate cell migration and invasion abilities. **(F)** Western blot assays were used to analyze the protein expression levels of apoptosis and anti-apoptosis proteins in GC cells (^∗^*P* < 0.05).

To investigate the ability of circ_0088300 in CAF-derived exosomes to promote migration and invasion in recipient GC cells, GC cells (SGC-7901 and BGC-823) were treated with different exosomes derived from CAFs, and exosomal circ_0088300 was overexpressed by transfecting pcDNA3/circ_0088300 into CAFs, while knockdown was achieved by transfecting si-circ_0088300 into CAFs. The data from the colony formation assays and EdU assays showed that circ_0088300 overexpression in CAF-derived exosomes significantly enhanced cell proliferation in SGC-7901 and BGC-823 cells, while circ_0088300 knockdown in CAF-derived exosomes decreased cell proliferation (Figures 5A–D). Moreover, the results of the Transwell assay revealed that CAF-derived exosomal circ_0088300 overexpression markedly enhanced migration and invasion in SGC-7901 and BGC-823 cells, while CAF-derived exosomal circ_0088300 knockdown significantly decreased migration and invasion (Figure 5E). In addition, Western blot assays showed that the expression of the anti-apoptosis protein Bcl-2 was upregulated and the apoptosis proteins Bax, caspase 3, and caspase 9 were downregulated when CAF-derived exosomal circ_0088300 was overexpressed; however, the knockdown of CAF-derived exosomal circ_0088300 decreased the expression of the anti-apoptosis protein Bcl-2 and increased the expression of apoptosis proteins (Figure 5F). These *in vitro* results proved that CAF-derived exosomal circ_0088300 overexpression could enhance malignant cell transformation *in vitro*.

### Exosomal circ_0088300 Acts as a Sponge for miR-1305 in GC Cells

**FIGURE 6:**
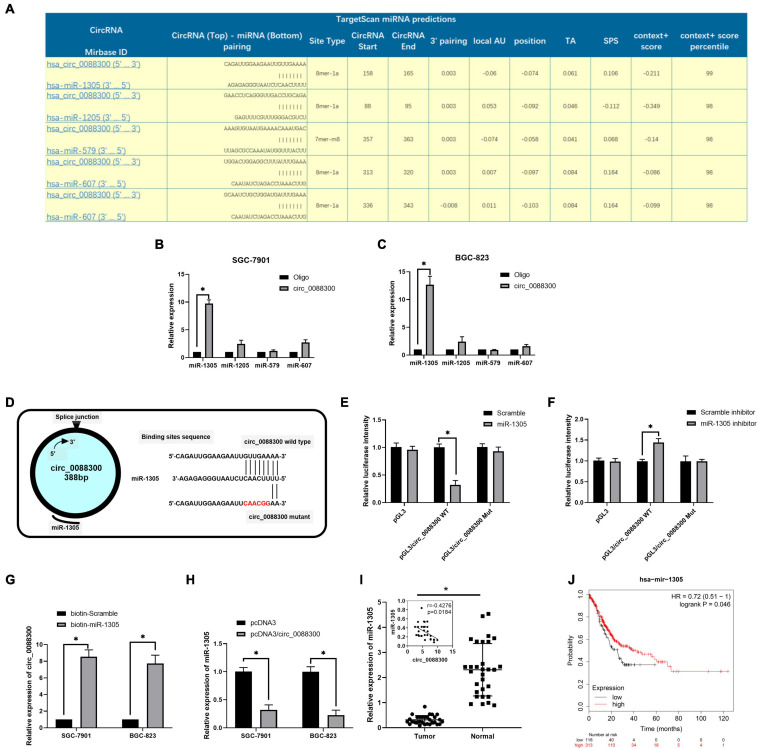
Exosomal circ_0088300 acts as a sponge for miR-1305 in GC cells. **(A)** The top five miRNAs predicted to be downstream of circ_0088300 by the circular RNA interactome. **(B,C)** RNA RIP experiments were performed in GC cells using a circ_0088300 probe or a control probe. The enrichment of potential target miRNAs was detected by qRT-PCR and normalized relative to the control (one-way analysis of variance, Dunnett’s test). **(D)** The putative binding sites of miR-1305 in the wild-type (WT) or mutated circ_0088300 sequences. **(E,F)** A luciferase reporter assay was used to detect the relationship between circ_0088300 and miR-1305 in GC cells. **(G)** A pull-down assay was used to analyze the binding of circ_0088300 and miR-1305. **(H)** qRT-PCR of miR-1305 expression in SGC-7901 and BGC-823 cells transfected with pcDNA3/circ_0088300 or controls. **(I)** the expression of miR-1305 in 30 GC tissues and the correlation analysis between circ_0088300 and miR-1305 in 30 GC tissues. **(J)** Survival curve of GC patients with high expression of miR-1305 and low expression of miR-1305, as generated by KM Plot online (^∗^*P* < 0.05).

CircRNAs have been found to act as miRNA sponges in tumor cells. To investigate the downstream miRNAs of circ_0088300, we identified predicted miRNA interactions via the circular interactome^2^ (Figure 6A and Supplementary Excel 3). hsa-miR-1305, hsa-miR-1205, hsa-miR-579, and hsa-miR-607 were predicted to bind with circ_0088300. We then purified the circ_0088300-associated RNAs using circ_0088300-specific probes and analyzed the five potential candidate miRNAs. We detected an enrichment of miR-1305 binding to circ_0088300 relative to that of the controls, while the other miRNAs demonstrated no enrichment in GC cells (Figures 6B,C). A luciferase reporter assay was used to further explore the interaction between circ_0088300 and miR-1305, and the results showed that, compared with transfection with a scrambled control, transfection with an miR-1305 mimic substantially attenuated the luciferase activity of wild type (WT) circ_0088300. Moreover, the miR-1305 mimic did not affect the luciferase activity of a circ_0088300 mutant. However, compared with the scrambled control, transfection with an miR-1305 inhibitor significantly improved the luciferase activity of wild type (WT) circ_0088300, while the luciferase activity of the circ_0088300 mutant was not affected (*P* < 0.01; Figures 6D–F). The RNA pull-down assay data showed that circ_0088300 was enriched in the biotin-labeled miR-1305 group (Figure 6G). The qRT-PCR results showed that the overexpression of circ_0088300 can decrease the expression of miR-1305 (Figure 6H). Moreover, we also observed that the expression of miR-1305 was downregulated in GC tissues compared with adjacent normal tissues, miR-1305 in GC tissues corresponded to clinicopathologic features (Supplementary Table 6), and the expression of miR-1305 in GC was negatively correlated with circ_0088300 in 30 paired GC tissues (Figure 6I). The survival curve also showed that GC patients who exhibited high circ_0088300 expression had a lower survival probability after diagnosis compared with patients who exhibited low expression of miR-1305 (Logrank *P* < 0.05; Figure 6J). These results indicated that exosomal circ_0088300 acts as a sponge for miR-1305 in GC cells.

### miR-1305 Inhibits Migration, Invasion, and Proliferation in GC Cells via JAK1 and STAT1 Downregulation

**FIGURE 7:**
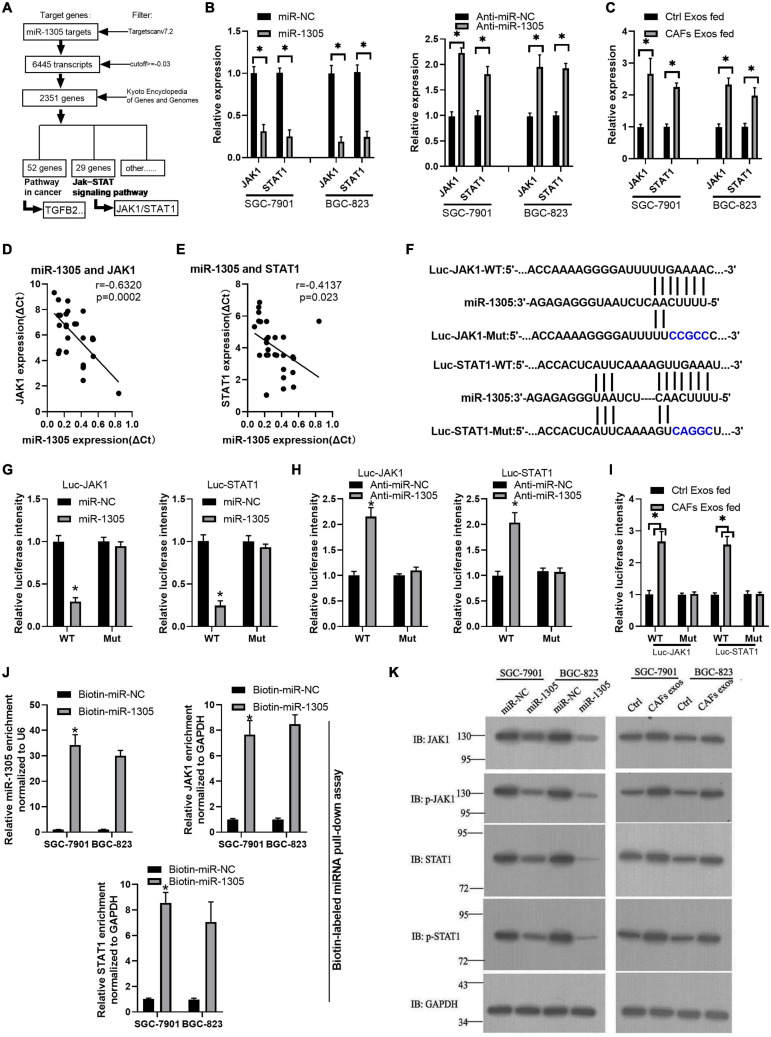
miR-1305 inhibits migration, invasion and proliferation in GC cells via JAK1 and STAT1 downregulation. **(A)** Analysis of genes and pathways downstream of miR-1305. **(B)** The expression of JAK1 and STAT1 in SGC-7901 and BGC-823 cells transfected by miR-1305 mimic or anti-miR-1305 by qRT-PCR; **(C)** The expression of JAK1 and STAT1 in SGC-7901 and BGC-823 cells treated with exosomes derived from normal cells and CAFs, as assessed by qRT-PCR. **(D)** Correlation analysis between JAK1 and miR-1305 in GC tissues (*n* = 30). **(E)** Correlation analysis between STAT1 and miR-1305 in GC tissues (*n* = 30). **(F)** Putative binding sites between miR-1305 and wild-type (WT) or mutated JAK1 (or STAT1) sequences. **(G)** Luciferase activity of JAK1 and STAT1 reporters in GC cells which transfected by miR-1305 or controls. **(H)** Luciferase activity of JAK1 and STAT1 reporters in GC cells that were transfected with anti-miR-1305 or controls. **(I)** Luciferase activity of JAK1 and STAT1 reporters in GC cells that were treated by exosomes derived from normal cells or CAFs. **(J)** RNA RIP experiments were performed with GC cells that were transfected with biotin-miR-1305 mimics or controls, and qRT-PCR analysis the enrichment of miR-1305, JAK1, and STAT1 in GC cells. **(K)** The protein level of JAK1 and STAT1 in SGC-7901 and BGC-823 cells transfected with miR-1305 mimics or control mimics, and CAF-derived exosomes or control exosomes (^∗^*P* < 0.05).

**FIGURE 8:**
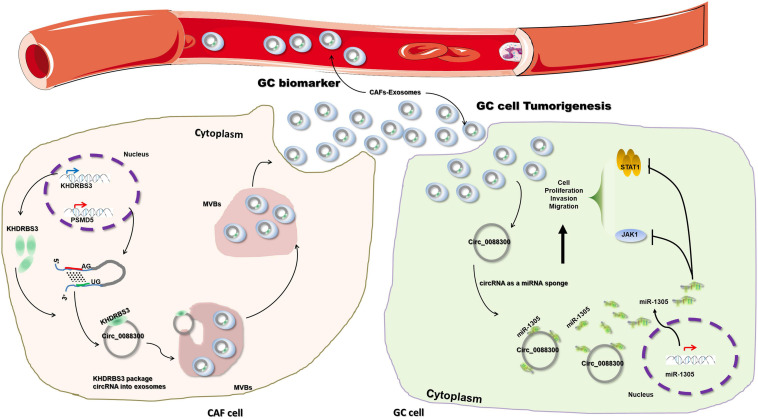
Schematic diagram illustrating the molecular mechanism of circ_0088300 in GC tumorigenesis. Exosomal circ_0088300 derived from cancer-associated fibroblasts acts as a sponge of miR-1305 and promotes gastric carcinoma cell tumorigenesis.

To further investigate the mechanism of miR-1305 in GC cell migration, invasion and proliferation, TargetScan v7.2 and KEGG analysis were used to screen the downstream genes of miR-1305 (Supplementary Excel 4). The JAK-STAT signaling pathway attracted our attention, and the data also showed that the JAK-STAT signaling pathway was more likely regulated by miR-1305 (Figure 7A and Supplementary Figure 7). A miR-1305 mimic and a miR-1305 inhibitor were transfected into GC cells to overexpress and knockdown miR-1305, respectively, and theqRT-PCR data showed that the expression of JAK1 and STAT1 was downregulated when miR-1305 was overexpressed, and the expression of JAK1 and STAT1 was upregulated when miR-1305 was knocked down (Figure 7B). Moreover, the expression of JAK1 and STAT1 in GC cells treated with CAF-derived exosomes was significantly higher than it was in normal cell-derived exosomes (Figure 7C). Additionally, the expression of JAK1 and STAT1 was negatively correlated with miR-1305 in GC tissues (Figures 7D,E). A luciferase reporter assay was performed to determine the interaction between miR-1305 and JAK1/STAT1, and the results showed that the miR-1305 mimic could attenuate the luciferase activity of JAK1/STAT1 with a wild type (WT) 3′-UTR, but it did not affect the luciferase activity of the JAK1/STAT1 with a mutant 3′-UTR. Transfection with the miR-1305 inhibitor also confirmed this effect (*P* < 0.05, Figures 7F–H). Furthermore, the luciferase activity of WT JAK1 (or STAT1) was increased when GC cells were treated with CAF-derived exosomes, while there was no effect on the JAK1 (or STAT1) mutant (Figure 7I). These data indicated that JAK1/STAT1 were regulated by miR-1305- and circ_0088300-enriched CAF-derived exosomes. Biotin-labeled miRNA pull-down assays also revealed the direct interaction between miR-1305 and JAK1/STAT1 (Figure 7J). Western blot analysis showed that JAK1 and STAT1 were regulated by miR-1305 or CAF-derived exosomes (Figure 7K). The data above suggest that miR-1305 inhibits migration, invasion and proliferation in GC cells via JAK1 and STAT1. Collectively, these results found that exosomal circ_0088300 derived from cancer-associated fibroblasts acts as a sponge of miR-1305 and promotes gastric carcinoma cell tumorigenesis (Figure 8).

## Discussion

### Identification of a Novel circRNA, circ_0088300 in Gastric Cancer Cells

GC patients have an unfavorable prognosis that is typically the result of a late diagnosis; additionally, the complex regulatory network of GC cell tumorigenesis is still a great barrier to GC treatment (Wei et al., 2020). To date, lots of circular RNAs have become a popular area of research in gastric cancer diagnosis and treatment (Ding et al., 2019; Huang et al., 2019; Zhu et al., 2019; Lu et al., 2020). For instance, circ-SERPINE2 promotes the development of GC (Liu et al., 2019); hsa_circ_0000520 acts as a patent biomarker for GC and is involved in GC tumorigenesis (Sun et al., 2018); and cirMC3 increases the progression of GC by sponging miRNA-296-5p with PTEN (Rong et al., 2019). Moreover, circRNAs have also been shown to be good biomarkers for cancer diagnosis (Meng et al., 2017; Yuan et al., 2018). In our study, we explored the RNA microarray of GC plasma to identify new targets. As a result, we found that a novel circRNA, circ_0088300, acts as an oncogene in GC. The results of clinical sample analysis provide further support for the crucial role of circ_0088300 in GC. Additionally, exosomes derived from the plasma of GC patients have abundant circ_0088300, which lays the foundation for new diagnostic methods for plasma-derived exosomes. Therefore, exosomal circ_0088300 might be a novel biomarker of GC diagnosis.

### The Specific Pattern of circ_0088300 Transfer Between CAFs and GC Cells

Previous studies have illustrated why the tumor microenvironment has long been suspected to play an important role in the initiation and progression of tumors (Hussain and Harris, 2007; Witz, 2008). CAFs are important constituents of the tumor stroma and have been linked to the invasion and proliferation of GC (Fuyuhiro et al., 2012; Kurashige et al., 2015). In the present study, we observed that cir_0088300 was more enriched in CAFs than in other cells, indicating that promoting tumorigenesis in CAFs might be related to circ_0088300 transfer. Recently, studies have demonstrated that CAF-derived exosomes boosted the metastasis and invasion of colorectal cancer (Hu et al., 2019) and endometrial cancer cells (Li et al., 2019); therefore, we hypothesized that exosomes derived from CAFs worked as carriers of circ_0088300, transferring it between CAFs and GC cells, and the results showed that GC cells cultured in CAF-CM expressed a higher level of circ_0088300; circ_0088300 expression in GC cells was significantly downregulated when CAF-derived exosomes were depleted physically or pharmacologically (GW4869), supporting our hypothesis.

However, the mechanism of circ_0088300 packaging into exosomes in CAFs is not clear. A previous study suggested that various defined RBPs could identify and sort RNAs with specific binding motifs into exosomes (Perez-Boza et al., 2018), such as hnRNPA2B1, which can bind and package miRNAs into exosomes based on conserved sequences named exosome motifs (Villarroya-Beltri et al., 2013), and could also specifically regulate the sorting of lncRNAs into exosomes (Qu et al., 2016). In this study, we screened the RBPs associated with circ_0088300 and verified that RBP KHDRBS3 specifically bound circ_0088300 through a particular sequence, and it regulated circ_0088300 packaging into exosomes. These findings might provide a mechanism of novel therapy through decreasing the level of circ_0088300 in GC cells, but cancer inhibitor factors in exosomes might also be eliminated from GC. Even so, the mechanism of sorting circRNAs into exosomes is still worth exploring further in the future.

### Support for the ceRNA Hypothesis in GC Progression

Competitive endogenous RNAs (ceRNAs) are transcripts that can regulate each other at the posttranscriptional level by competing for shared miRNAs; these processes link the function of protein-coding mRNAs with that of non-coding RNAs, such as microRNAs, long non-coding RNAs, pseudogenic RNAs and circRNAs (Tay et al., 2014; Qi et al., 2015). CeRNAs, such as lncRNAs, circRNAs and pseudogenes, have been proven to be involved in cancer progression by acting as miRNA “sponges” by sharing miRNA response elements (MREs) to control gene expression (Kristensen et al., 2018; Zhao et al., 2018; Shang et al., 2019). In the present study, we hypothesized that circ_0088300 acts as a sponge of miRNA, which inhibits GC cell tumorigenesis, and then we predicted the miRNAs binding to circ_0088300. We observed that miR-1305 could inhibit GC progression and that the expression of miR-1305 in GC was negatively correlated with circ_0088300, which supported the idea that exosomal circ_0088300 acts as a sponge for miR-1305 in GC cells. Furthermore, the downstream signaling pathways of circ_0088300/miR-1305 and JAK/STAT were assessed. To date, exosomal circ_0088300 has been verified to promote GC cell tumorigenesis via the circ_0088300/miR-1305/JAK/STAT axis.

However, some limitations of this study should be considered when interpreting the results and should be addressed in future research. First, although the exosome secretion inhibitor GW4869 significantly alleviated CAF-mediated tumorigenesis promotion in GC cells, *in vivo* experiments should be performed. Second, we need to further explore other mechanisms of circRNA packaging into exosomes and find more potential targets to block GC cell tumorigenesis initiated by the transfer of CAF exosomes. Thirdadditional evidence from a larger number of patients is still needed to verify that exosomal circ_0088300 could act as a biomarker of GC diagnosis. Finally, while we reveal circ_0088300 act as an oncogene in GC, other circRNAs have been reported to suppress the progression of gastric cancer (Zhang et al., 2017; Luo et al., 2020). So, it could act as dual roles in GC progress, which may depend on the nature of the circular RNAs. Of course, more and more circRNAs should be revealed in the further study.

In summary, we illustrated that circ_0088300 is substantially upregulated in human GC tissues and plasma, and CAF-derived exosomal circ_0088300 acts as a sponge of miR-1305 to promote the proliferation, migration and invasion of GC cells. We also demonstrated that circ_0088300 can promote the tumorigenesis of GC cells through the JAK/STAT signaling pathway. Circ_0088300 has the potential of being a biomarker of GC diagnosis, and inhibition of circ_0088300 could be a novel therapeutic strategy for GC in the future.

## Data Availability Statement

The original contributions presented in the study are included in the article/Supplementary Material, further inquiries can be directed to the corresponding author/s.

## Ethics Statement

The studies involving human participants were reviewed and approved by the Ethics Committee of The First Affiliated Hospital of Soochow University. The patients/participants provided their written informed consent to participate in this study. The animal study was reviewed and approved by The Ethics Committee of The First Affiliated Hospital of Soochow University.

## Author Contributions

HS, SH, and LG contributed to study concept and design, acquisition, analysis, interpretation of data, and drafting of the manuscript. MQ and XX contributed to data collections and manuscript review. HS, SH, LG, XG, LJ, HH, and JF contributed to drafting of the manuscript. HS supervised the study. All authors read and approved the final manuscript.

## Conflict of Interest

The authors declare that the research was conducted in the absence of any commercial or financial relationships that could be construed as a potential conflict of interest.

## Footnotes

1 http://rbpdb.ccbr.utoronto.ca/

2 https://circinteractome.nia.nih.gov/index.html

## Abbreviations

CAFs: cancer-associated fibroblasts
ceRNA: competing endogenous RNA
circRNA: circular RNA
GC: gastric carcinoma
miRNA: microRNA.
