# Supplementary material for: Exosomal circ_0088300 Derived From Cancer-Associated Fibroblasts Acts as a miR-1305 Sponge and Promotes Gastric Carcinoma Cell Tumorigenesis
Source: Front Cell Dev Biol. 2021 May 26;9:676319. doi: 10.3389/fcell.2021.676319 (PMC8188357; doi:10.3389/fcell.2021.676319)
Supplement: Supplementary file 1 [file Table_1.DOCX]

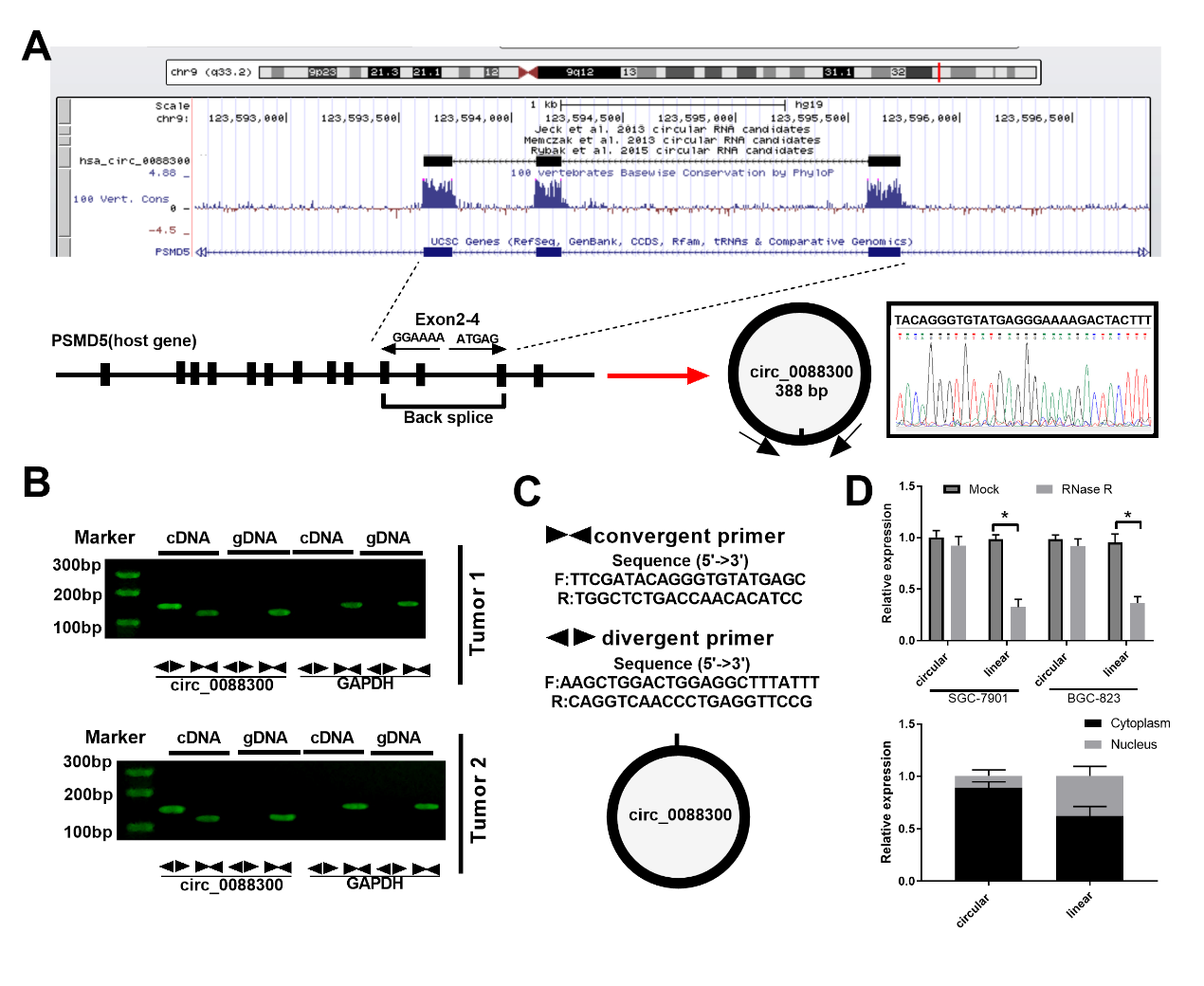


**Fig. S1 Identification of circ_0088300 in GC cells.** A) Schematic representation of circ_0088300 formation and the sanger sequence of circ_0088300 PCR products by using convergent primers; B-C) the convergent primer and disvergent primer of circ_0088300 were designed, and relative PCR products were subjected to Agarose gel analysis. Divergent primers could produce circRNAs in cDNA but not in genomic DNA (gDNA); convergent primers could produce cDNA and gDNA; D) the expression of circular and liner forms of PSMD5 mRNAs in GC cells treated by RNase R or controls. And the expression of circular(circ_0088300) or linear form of PSMD5 mRNAs in cytoplasm and nucleus were analysis by qRT-PCR.


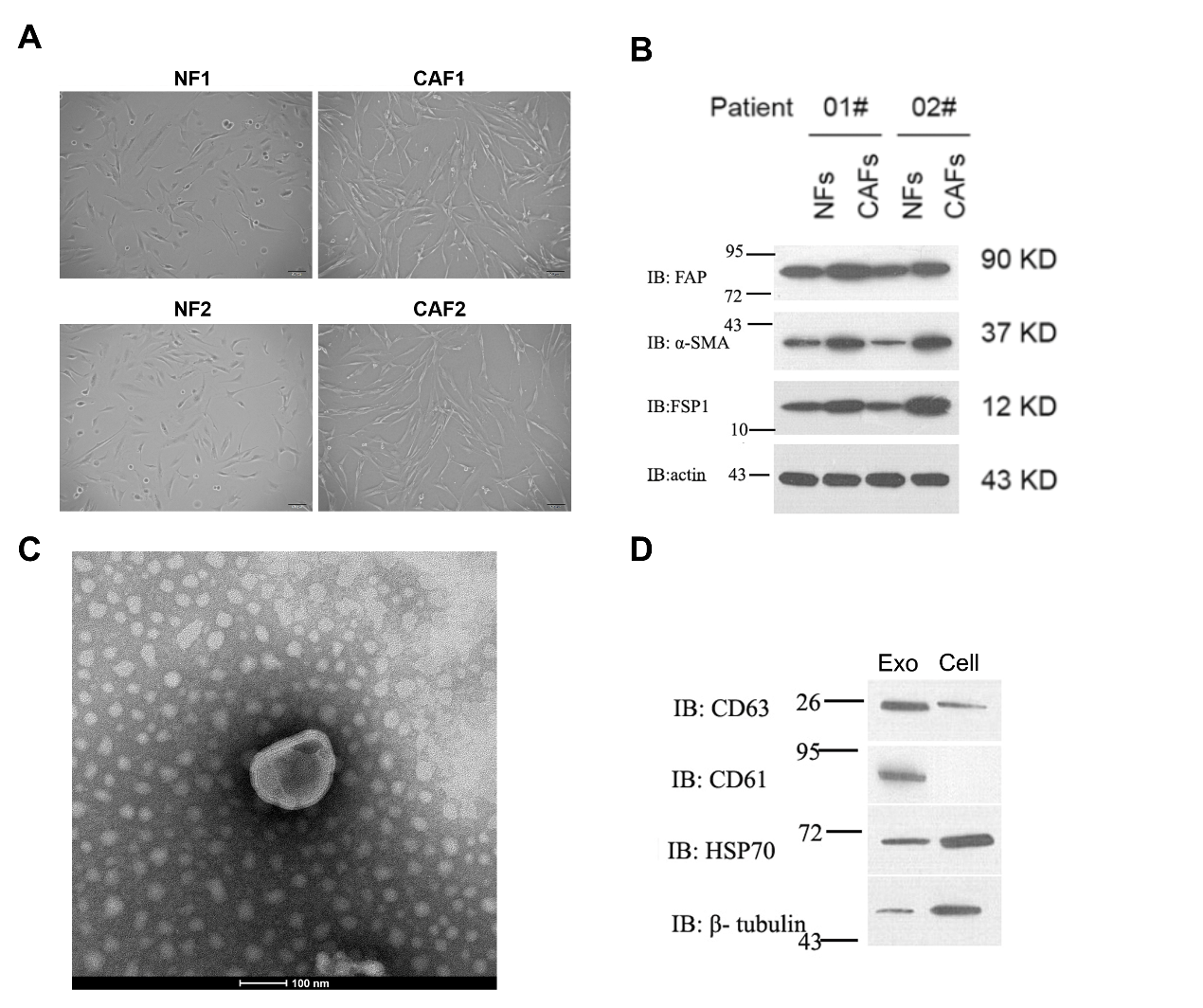


**Fig. S2 Identification of NFs, CAFs and exosomes from CAF cells.** A) The morphology in optical microscope of NFs and CAFs from two patients; B) the expression of special protein in NFs and CAFs by Western blot; C) the morphology in transmission electron microscope of CAFs-derived exosomes; D) the expression of exosomes characteristic protein in CAFs and CAFs-derived exosomes.


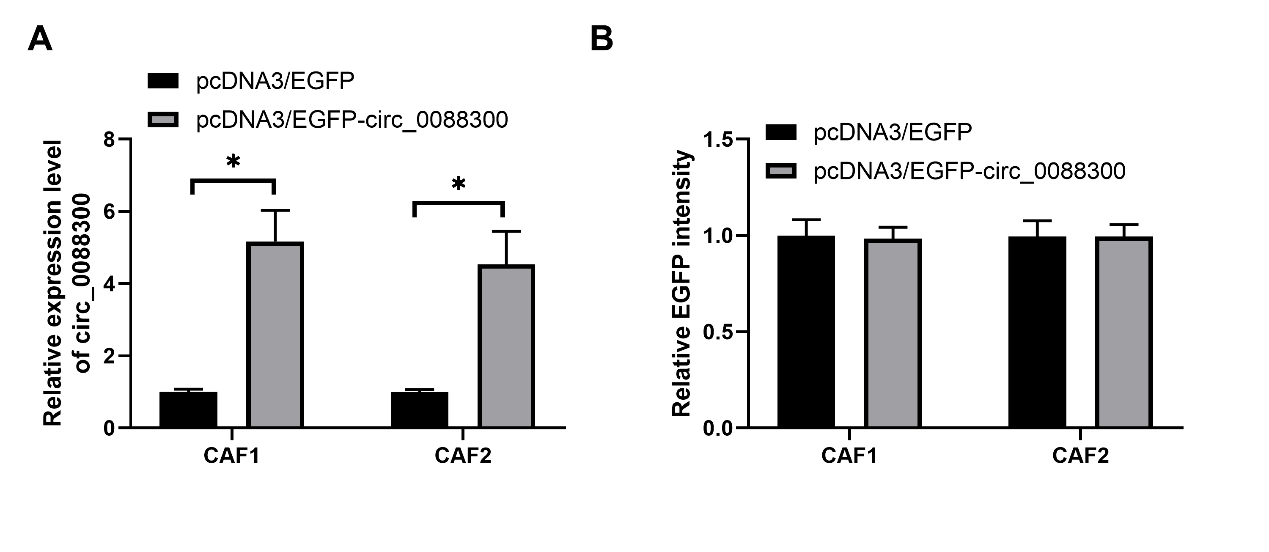


**Fig. S3 Over-expression of pcDNA3/EGFP-circ_0088300 effecience in CAFs.** A) the expression of circ_0088300 in CAFs transfected by pcDNA3/EGFP-circ_0088300 or pcDNA3/EGFP; B) the intensity of EGFP in CAFs transfected by pcDNA3/EGFP-circ_0088300 or pcDNA3/EGFP.


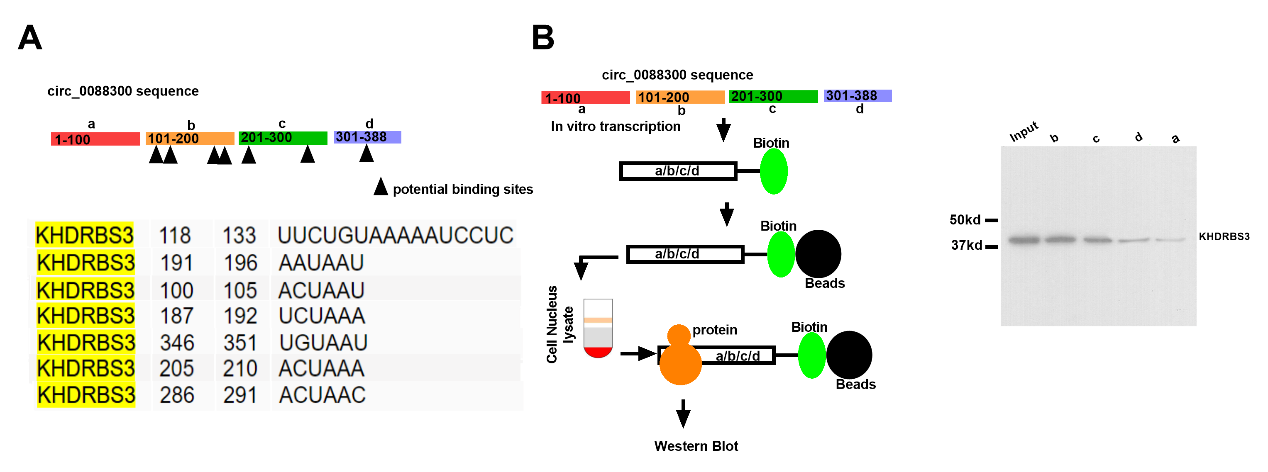


**Fig. S4 The interaction between circ_0088300 and KHDRBS3 was validated by RNA immunoprecipitation (RIP) in CAF cells.** A) the circ_0088300 binding squences and sites with KHDRBS3 predicted by RBPDB; B) RNA pull-down assay to verify the interaction between circ_0088300 and KHDRBS3. Western blot analysis were performed to validated the KHDRBS3 in different groups. Input as a normal control.


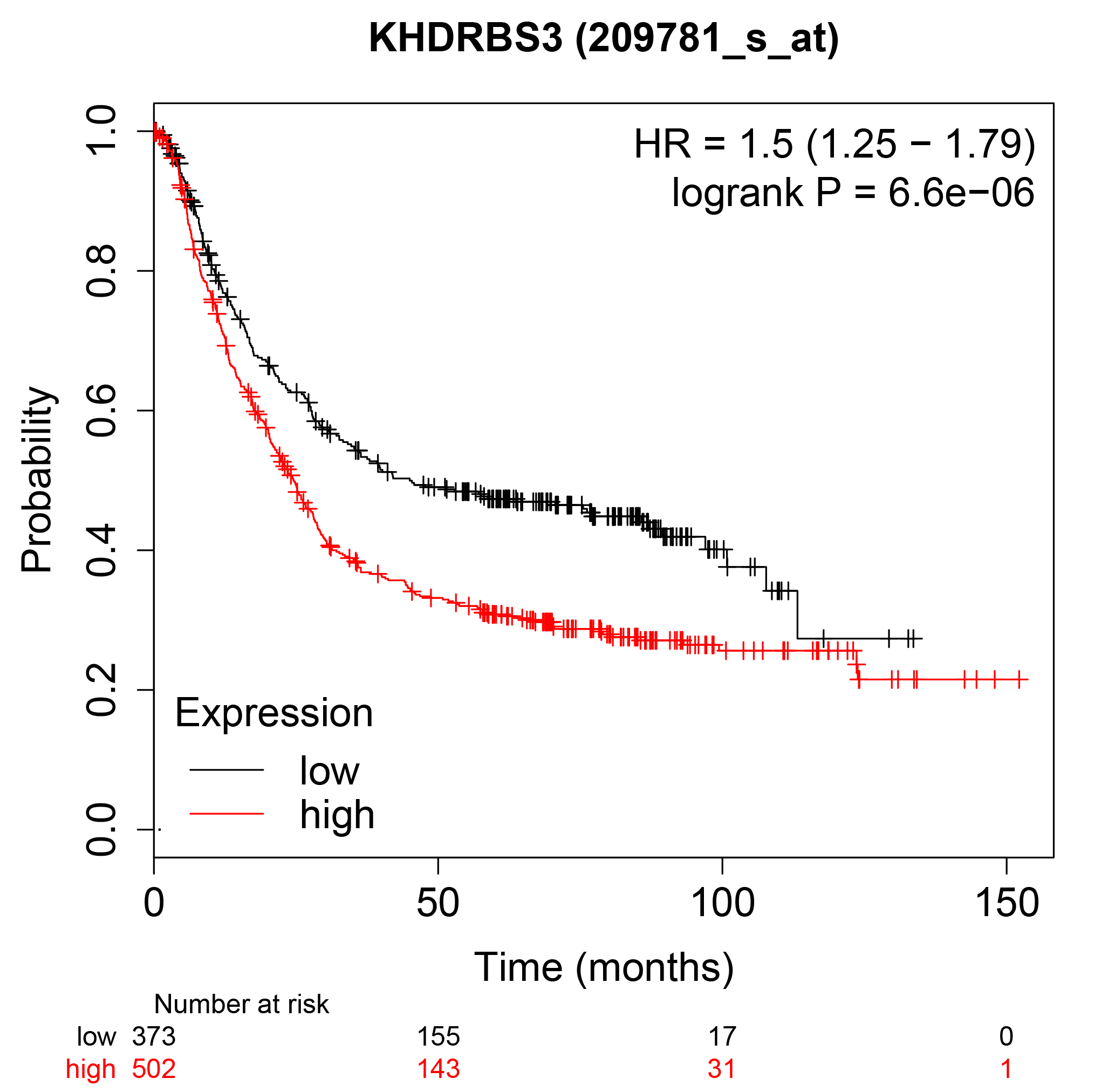


**Fig. S5 Survival curve of GC patients with high expression of KHDRBS3 and low expression of KHDRBS3 analysis by KM Plot bioinformatic on line tool.**


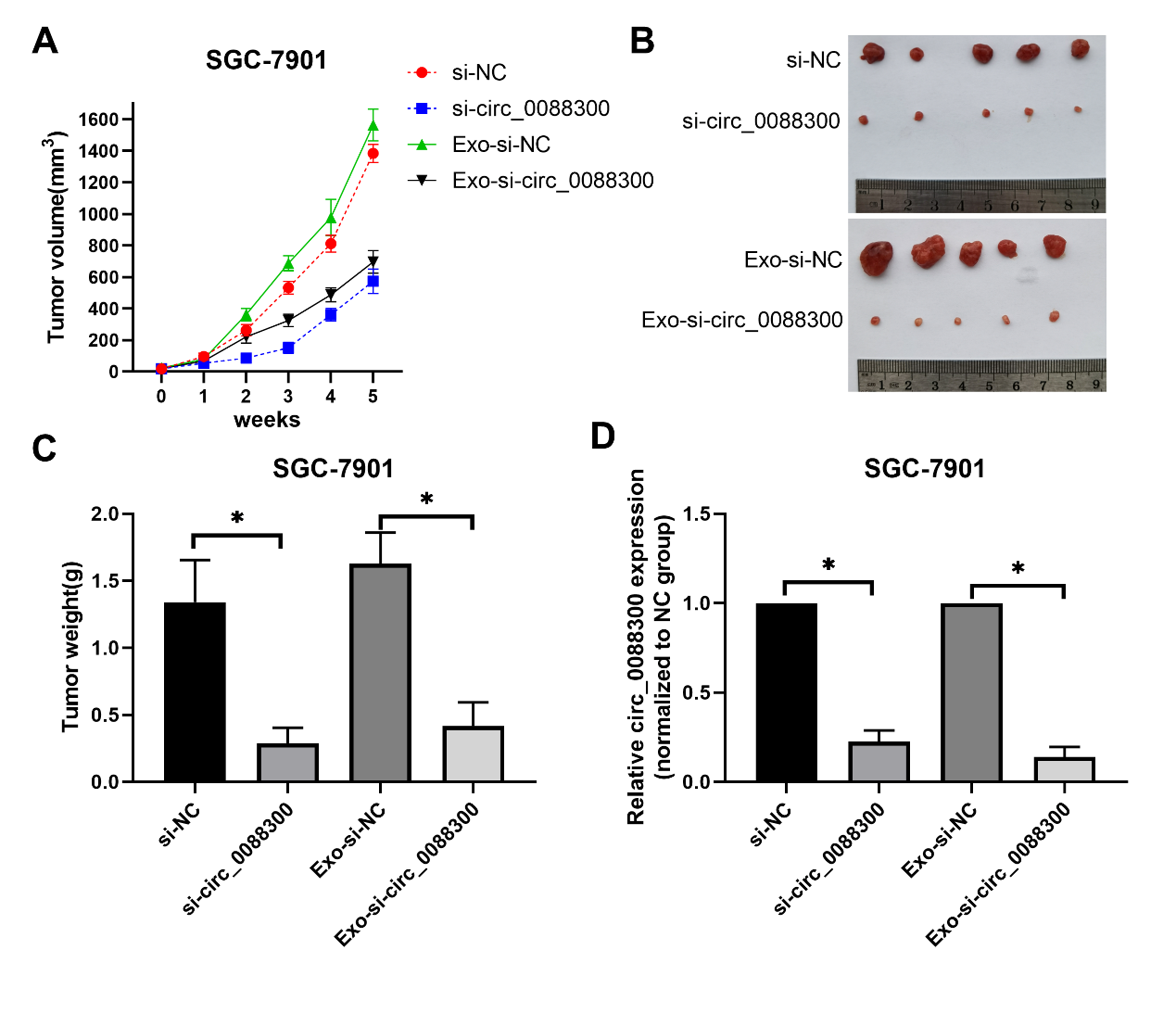


**Fig. S6 circ_0088300 enhance GC cell tumorigenesis *in vivo*.** A) Tumor volume of the SGC-7901 tumorigenesis assay in nude mice; B) the tumor morphology of tumorigenesis assay in nude mice after 5 weeks; C) tumor weight in each group after the nude mice were sacrificed at end time point; D) qRT-PCR analysis were performed to detect the expression of circ_0088300 in each group (normalized to si-NC).


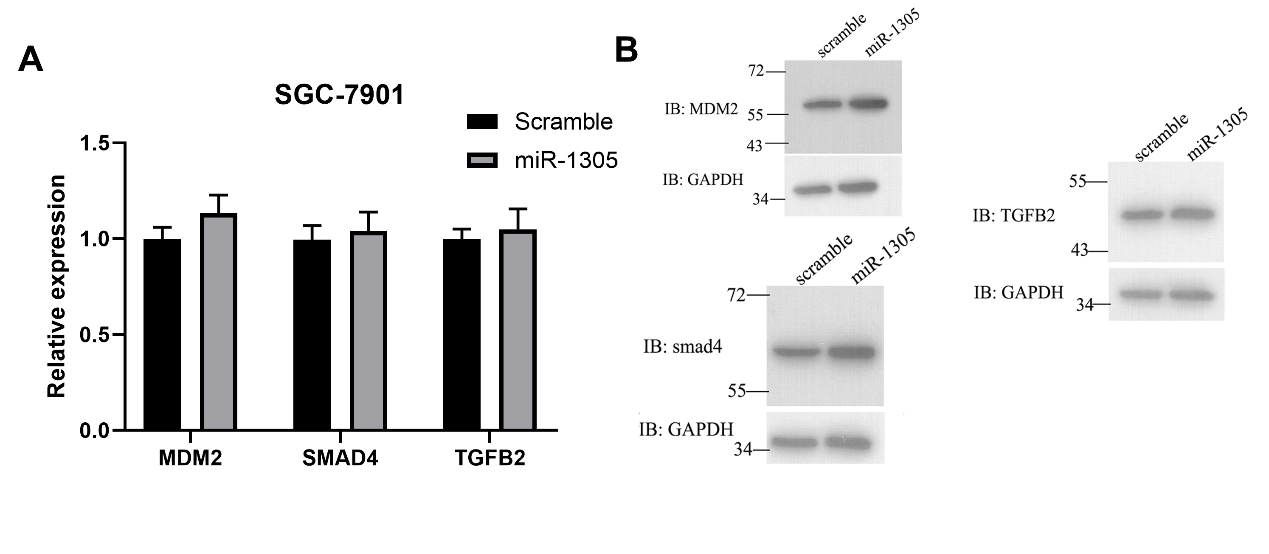


**Fig. S7 The effect of miR-1305 on the expression of TGFB2, MDM2 and Smad4 in GC cells.** A) qRT-PCR analysis the expression of MDM2, SMAD4, and TGFB2 in SGC-7901 cells transfected by miR-1305 mimics or control mimics; B) Western blot assay of the expression of MDM2, SMAD4, and TGFB2 in transfected SGC-7901 cells.
