# Supplementary material for: Exosomal circ_0088300 Derived From Cancer-Associated Fibroblasts Acts as a miR-1305 Sponge and Promotes Gastric Carcinoma Cell Tumorigenesis
Source: Front Cell Dev Biol. 2021 May 26;9:676319. doi: 10.3389/fcell.2021.676319 (PMC8188357; doi:10.3389/fcell.2021.676319)
Supplement: Supplementary file 2 [file Table_2.DOCX]

**Co-culture assay**

The co-culture assay was performed using transwell membranes (0.8μm, Corning, USA) in a 24-well plate. CAFs were pretreated with GW4869 (Sigma, USA) or DMSO (Sigma, USA) for 24 h. The co-culture was sustained for 3 days, and CAFs on the permeable membranes, then GC cells (5×10^4^ cells) below the membranes were collected for RNA extraction or further experiments.

**Isolation and purification of exosomes**

Exosomes were isolated from CAFs supernatant. The medium was then collected and centrifuged at 300×g for 10 min, followed by centrifugation at 2000 × g for 20 min at 4°C. After centrifugation, the supernatant was filtered using a 0.22-µm filter to remove dead cells and large cellular debris. Afterwards, Small cellular debris was divided by centrifugation at 10,000g for 30 minutes, and then the supernatants were recentrifuged at 100,000g for 70 minutes. Finally, 100µL PBS solution was used to resuspend the exosome on the bottom of ultracentrifuge tube. Exosomes were quantitated by measuring the total protein concentration, detected by bicinchoninic acid assay (BCA; Thermo Fisher).

**Cell transfection**

Plasmids was transfected by using Lipofectamine 3000 reagent (Invitrogen, USA) according to the manufacturer’s protocols. Transfection of siRNA, miRNA mimics or inhibitors (GenePharma, China) was performed using Lipofectamine RNAiMAX (Invitrogen, USA) at a final concentration of 100 nM. Sequences of siRNA against specific targets in this study were listed in Table S2.

**RNA binding protein immunoprecipitation (RIP) assay**

RIP assay was completed by using an EZMagna RIP kit (Millipore, USA). CAFs were collected and lysed with RIP lysis buffer. CAFs lysates (100 μl) were treated with RIP buffer and were incubated with Proteinase K and magnetic beads conjugated with anti- KHDRBS3 antibody or control (IgG). Next, the immunoprecipitated RNA was extracted. The isolated miRNA wasreverse transcribed and then analyzed by qPCR.

**Biotin miRNA pull-down assay**

The NE-PERTM nuclear and cytoplasmic extraction reagents (Thermo Scientific, USA) were used to separate and prepare cytoplasmic and nuclear extracts from CAFs. The nuclear, cytoplasmic, or exosomal lysates of CAFs were incubated overnight at 4 °C with 100 pmol of synthetic single-stranded circ_0088300 or mutated circ_0088300 oligonucleotides containing a biotin modification attached to the 5′ end via a spacer arm (Sigma-Aldrich, USA). Washed streptavidin agarose beads (Invitrogen, USA) were added to each binding reaction, which was further incubated at 4 °C for 4 h. Precipitates were washed five times and boiled in SDS buffer, followed by western blotting analysis. Biotinylated poly(G) (5′-GGGGGGGGGGGGGGGGGGGGG-3′) was used as a negative control. List of probe sequences for RNA pulldownin this study were listed in Table S3.

**Western blot analysis**

Protein lysates were extracted from SGC-7901 and GES-1 cells by incubating with RIPA lysis and extraction buffer (Thermo Fisher, USA). Protein concentration was detected by BCA kit (Thermo Fisher, USA). Equal amounts of proteins were separated by sodium dodecyl sulfate polyacrylamide gel electrophoresis, transferred to polyvinylidene difluoride membranes (Millipore, USA), and incubated overnight at 4°C with primary antibodies followed by blocking with bovine serum albumin (5%, v/v). The primary antibodies included Bax (1:1000, rabbit IgG; Abcam, USA), Bcl-2 (1:1000, rabbit IgG; Abcam, USA), caspase-3 (1:2000, rabbit IgG; Abcam, USA),Actin (1:1000, Abcam, USA). Membranes were then incubated with the secondary antibody (1:4000, Proteintech, China) for 120 min at RT. Reacting bands were visualized using enhanced chemiluminescence reagent (Proteintech, China) and the density of the protein bands was semi-quantified using the software Image J.

**Clonogenic assay**

We seeded pcDNA3, pcDNA3/circ_0088300, si-NC, and si-circ_0088300 SGC-7901and BGC-823 cells in a 6-well plate (500 cells per well) for 2 weeks. All cells in plates were then fixed in 2 mL of 4% paraformaldehyde for 30 mins and stained with crystal violet for 30 mins.

**Transwell assay**

The protocols of the migration and invasion assay were similar, the migration assay and invasion assay used the different type of membrane: The membrane in the upper transwell chamber for the invasion assay was a Matrigel-coated membrane (BD Biosciences), while that for the migration assay was a normal membrane. Transwell assay inserts (0.8μm, Corning, USA) were placed in a 24-well plate. 500 μl of serum-free RPMI 1640 with 10% FBS was placed in the bottom chamber. And then, we seeded 1×10^4^ SGC-7901 or BGC-823 cells in 200 μl of RPMI 1640 in the upper chamber. After 24 to 48 h, we used methanol to fix the cells within the membrane and stained them with crystal violet. Finally, the cells were observed by microscope.

**Luciferase reporter assay**

Potential binding sites were predicted by the TargetScan database (<http://www.targetscan.org>). GC cells (1 × 105 cells/well) were added to a 24-well plate and co-transfected with 200 ng pGL3/circ_0088300 WT or pGL3/circ_0088300 Mut and 80 nmol miRNA mimics or miRNA inhibitor. After 48 h of transfection, the cells were harvested, and luciferase activities were measured with the Dual-Luciferase Reporter Assay System (Promega, USA).
