# Supplementary material for: Exosomal circ_0088300 Derived From Cancer-Associated Fibroblasts Acts as a miR-1305 Sponge and Promotes Gastric Carcinoma Cell Tumorigenesis
Source: Front Cell Dev Biol. 2021 May 26;9:676319. doi: 10.3389/fcell.2021.676319 (PMC8188357; doi:10.3389/fcell.2021.676319)
Supplement: Supplementary file 3 [file Table_3.DOCX]

Table S1 List of primers for qPCR

| Gene | Sequence (5’-3’) |
| --- | --- |
| Circ_0088300 | F: AAGCTGGACTGGAGGCTTTATTT |
|  | R: CAGGTCAACCCTGAGGTTCCG |
| miR-1305 | F: CTCAACTGGTGTCGTGGAGTCGGCAATTCAGTTGAGCAGTAGAG |
|  | R: ACACTCCAGCTGGGGTGTTGAAACAATCT |
| GAPDH | F:T GTTCGTCATGGGTGTGAAC |
|  | R: ATGGCATGGACTGTGGTCAT |
| U6 | F: CTCGCTTCG GCAGCACA |
|  | R: AACGCTTCACGAATTTGCGT |
| PSMD5 linear mRNA | F: TTCGATACAGGGTGTATGAGC |
|  | R: TGGCTCTGACCAACACATCC |
| miR-1205 | F: CTGCAGGGTTTGCTTTGAGG |
|  | R: CTCCAGAACAGGGTTGACAGG |
| miR-579 | F: CGTGCCGTTCATTTGGTATAAAC |
|  | R: GAGCAGGGTCCGAGGT |
| miR-607 | F: ACACTCCAGCTGGGCTAGTGGTTCAAATCCAGA |
|  | R: CTCAACTGGTGTCGTGGAGTCGGCAATTCAGTTGAGGTTATAGA |
| KHDRBS3 | F: GAGGAGAAGTACCTGCCCGAG |
|  | R: ATAGTAATCAGCACCACTTTGGGC |
| JAK1 | F: ACAATTGGCATTCATTTTCCTG |
|  | R: CCTGGGCCCAAACTTCCTA |
| STAT1 | F: GATCTCTAACGTCTGTCAGCTG |
|  | R: GAGGTCCAGGAT TCCTTCGATC |

Table S2. List of sequences for siRNAs

| siRNA | Sequence(5' -3') |
| --- | --- |
| Si-ZFP36#1 | sense: GCGCUACAAGACUGAGCUAUG |
|  | antisense: UAGCUCAGUCUUGUAGCGCGA |
| Si-ZFP36#2 | sense: AGACGGAACUCUGUCACAAGU |
|  | antisense: UUGUGACAGAGUUCCGUCUUG |
| Si-KHDRBS3#1 | sense: GAAGAGUGGACUAACUCAAGA |
|  | antisense: UUGAGUUAGUCCACUCUUCUU |
| Si-KHDRBS3#2 | sense: GCUGGGACAGAAAGUGUUAAU |
|  | antisense: UAACACUUUCUGUCCCAGCUU |
| Si-QKI#1 | sense: GAGCAGAAAUCAAAUUGAAGA |
|  | antisense: UUCAAUUUGAUUUCUGCUCUG |
| Si-QKI#2 | sense: GGAAAGACAUGUACAAUGACA |
|  | antisense: UCAUUGUACAUGUCUUUCCGU |
| Si-PABPC1#1 | sense: CGGUGUUCCAACUGUUUAA |
|  | antisense: UUAAACAGUUGGAACACCGGU |
| Si-PABPC2#2 | sense: GCCUGCUGUUCAUGUACAAGG |
|  | antisense: UUGUACAUGAACAGCAGGCUG |
| Si-EIF4B#1 | sense: GGAAGUGAGUCAUCACAAACU |
|  | antisense: UUUGUGAUGACUCACUUCCUG |
| Si-EIF4B#2 | sense: AGUUCAGUUCUGCAAGCAAGU |
|  | antisense: UUGCUUGCAGAACUGAACUUG |
| Si-circ_0088300#1 | sense: CAAAGUAGUCUUUUCCCUCAUA |
|  | antisense: UGAGGGAAAAGACUACUUUGGU |
| Si-circ_0088300#2 | sense: UCUUUUCCCUCAUACACCCUGUAU |
|  | antisense: ACAGGGUGUAUGAGGGAAAAGAUG |

Table S3. List of probe sequences for RNA pulldown

| Gene | Sequence (5’-3’) |
| --- | --- |
| miR-1305 (5’biotin) | UUUUCAACUCUAAUGGGAGAGAUCUCCCAUUAGAGUUGAGGGUU |
| miR-nc (5’biotin) | UUCUCCGAACGUGUCACGUTTACGUGACACGUUCGGAGAATT |
| Circ_0088300-(5’biotin) | UACAGGGUGTAUGAGGGAAAAGACUACUUUGUGUGUAUCCAUUCUGGAGAGAUUGCUCCA |
| Oligo (5’biotin) | GGUACUGGAUAUAGUACAGAUCCAGUCCUUCUGUGGA |

Table-S4. Relationship between tissue circ_0088300 level and clinicopathologic features in GC.

| Clinicopathologic Characteristics | No. of patients | Circ_0088300 | | *P* value |
| --- | --- | --- | --- | --- |
|  |  | High | Low |  |
| **Age (year)** |  |  |  |  |
| >60 | 45 | 22 (48.9%) | 23 (51.1%) | 0.328 |
| ≤60 | 15 | 9 (60.0%) | 6 (40.0%) |  |
| **Gender** |  |  |  |  |
| Male | 42 | 23 (54.8%) | 19 (45.2%) | 0.591 |
| Female | 18 | 10 (55.6%) | 8 (44.4%) |  |
| **Tumor size (cm)** |  |  |  |  |
| <3 | 28 | 11 (39.3%) | 17 (60.7%) | 0.098 |
| ≥3 | 32 | 19 (59.4%) | 13 (40.6%) |  |
| **Differentiation grade** |  |  |  |  |
| Well/moderately | 21 | 10 (47.6%) | 11 (52.4%) | 0.284 |
| Poorly/undifferentiated | 39 | 23 (59.0%) | 16 (41.0%) |  |
| **Invasion** |  |  |  |  |
| T0-T2 | 23 | 8 (34.8%) | 15 (65.2%) | 0.036* |
| T3-T4 | 37 | 23 (62.2%) | 14 (37.8%) |  |
| **Lymph nodes** |  |  |  |  |
| N0-N2 | 39 | 13 (33.3%) | 26 (66.7%) | 0.005** |
| N3 | 21 | 15 (71.4%) | 6 (28.6%) |  |
| **Nerve invasion** |  |  |  |  |
| None | 48 | 20 (66.7%) | 28 (33.3%) | 0.110 |
| Yes | 12 | 8 (66.7%) | 4 (33.3%) |  |
| **Vascular invasion** |  |  |  |  |
| None | 45 | 19 (42.2%) | 26 (57.8%) | 0.089 |
| Yes | 15 | 10 (66.7%) | 5 (33.3%) |  |
| **TNM stage** |  |  |  |  |
| I & II | 29 | 11 (37.9%) | 18 (62.1%) | 0.035* |
| III & IV | 31 | 20 (64.5%) | 11 (35.3%) |  |
| Ki67 (+%) |  |  |  |  |
| ≤60 | 23 | 9 (39.1%) | 14 (60.9%) | 0.046* |
| >60 | 37 | 24 (64.9%) | 13 (35.1%) |  |

**P*<0.05, TNM stage: Pathologic tumor, node, metastasis stage.

Table-S5. Relationship between plasma exosomal circ_0088300 level and clinicopathologic features in GC.

| Clinicopathologic Characteristics | No. of patients | Circ_0088300 | | *P* value |
| --- | --- | --- | --- | --- |
|  |  | High | Low |  |
| **Age (year)** |  |  |  |  |
| >60 | 45 | 24 (53.3%) | 21 (46.7%) | 0.618 |
| ≤60 | 15 | 8 (53.3%) | 7 (46.7%) |  |
| **Gender** |  |  |  |  |
| Male | 42 | 20 (47.6%) | 22 (52.4%) | 0.250 |
| Female | 18 | 11 (61.1%) | 7 (38.9%) |  |
| **Tumor size (cm)** |  |  |  |  |
| <3 | 28 | 15 (53.6%) | 13 (46.4%) | 0.589 |
| ≥3 | 32 | 17 (53.1%) | 15 (46.4%) |  |
| **Differentiation grade** |  |  |  |  |
| Well/moderately | 21 | 9 (42.9%) | 12 (57.1%) | 0.132 |
| Poorly/undifferentiated | 39 | 24 (61.5%) | 15 (38.5%) |  |
| **Invasion** |  |  |  |  |
| T0-T2 | 23 | 9 (39.1%) | 14 (60.9%) | 0.046* |
| T3-T4 | 37 | 24 (64.9%) | 13 (35.1%) |  |
| **Lymph nodes** |  |  |  |  |
| N0-N2 | 39 | 11 (28.2%) | 28 (71.8%) | 0.012* |
| N3 | 21 | 13 (61.9%) | 8 (38.1%) |  |
| **Nerve invasion** |  |  |  |  |
| None | 48 | 17 (35.4%) | 31 (64.6%) | 0.132 |
| Yes | 12 | 7 (58.3%) | 5 (41.7%) |  |
| **Vascular invasion** |  |  |  |  |
| None | 45 | 20 (44.4%) | 25 (55.6%) | 0.228 |
| Yes | 15 | 9 (60.0%) | 6 (40.0%) |  |
| **TNM stage** |  |  |  |  |
| I & II | 29 | 12 (41.4%) | 17 (58.6%) | 0.020* |
| III & IV | 31 | 22 (71.0%) | 9 (29.0%) |  |
| Ki67 (+%) |  |  |  |  |
| ≤60 | 23 | 9 (45.5%) | 14 (54.5%) | 0.017* |
| >60 | 37 | 26 (70.3%) | 11 (29.7%) |  |

**P*<0.05, ***P*<0.01, TNM stage: Pathologic tumor, node, metastasis stage.

Table-S6. Relationship between tissue miR-1305 level and clinicopathologic features in GC.

| Clinicopathologic Characteristics | No. of patients | miR-1305 | | *P* value |
| --- | --- | --- | --- | --- |
|  |  | High | Low |  |
| **Age (year)** |  |  |  |  |
| >60 | 45 | 20 (44.4%) | 25 (55.6%) | 0.116 |
| ≤60 | 15 | 10 (66.7%) | 5 (33.3%) |  |
| **Gender** |  |  |  |  |
| Male | 42 | 25 (59.5%) | 17 (40.5%) | 0.497 |
| Female | 18 | 10 (55.6%) | 8 (44.4%) |  |
| **Tumor size (cm)** |  |  |  |  |
| <3 | 28 | 17 (60.7%) | 11 (39.3%) | 0.146 |
| ≥3 | 32 | 14 (43.8%) | 18 (56.3%) |  |
| **Differentiation grade** |  |  |  |  |
| Well/moderately | 21 | 16 (76.2%) | 5 (23.8%) | 0.015* |
| Poorly/undifferentiated | 39 | 17 (43.6%) | 22 (56.4%) |  |
| **Invasion** |  |  |  |  |
| T0-T2 | 23 | 16 (69.6%) | 7 (30.4%) | 0.016* |
| T3-T4 | 37 | 14 (37.8%) | 23 (62.2%) |  |
| **Lymph nodes** |  |  |  |  |
| N0-N2 | 39 | 16 (41.0%) | 23 (59.0%) | 0.382* |
| N3 | 21 | 7 (33.3%) | 14 (66.7%) |  |
| **Nerve invasion** |  |  |  |  |
| None | 48 | 29 (60.4%) | 19 (39.6%) | 0.087 |
| Yes | 12 | 4 (33.3%) | 8 (66.7%) |  |
| **Vascular invasion** |  |  |  |  |
| None | 45 | 27 (60.0%) | 18 (40.0%) | 0.147 |
| Yes | 15 | 6 (40.0%) | 9 (60.0%) |  |
| **TNM stage** |  |  |  |  |
| I & II | 29 | 16 (55.2%) | 13 (44.8%) | 0.063 |
| III & IV | 31 | 10 (32.3%) | 21 (67.7%) |  |
| Ki67 (+%) |  |  |  |  |
| ≤60 | 23 | 15 (65.2%) | 8 (34.8%) | 0.013* |
| >60 | 37 | 12 (32.4%) | 25 (67.6%) |  |

**P*<0.05, TNM stage: Pathologic tumor, node, metastasis stage.
